# Designing a Roadmap for Health Technology Assessment Implementation in Algeria

**DOI:** 10.7759/cureus.65558

**Published:** 2024-07-28

**Authors:** Meriem Hedibel, Fatma-Zohra Ghanassi, Kareem A El-Fass, Ahmad N Fasseeh, Sherif Abaza, Zoltán Kaló

**Affiliations:** 1 Pharmaceutics and Industrial Pharmacy Research Laboratory, Faculty of Pharmacy, University of Algiers, Algiers, DZA; 2 Health Economics, Syreon Middle East, Alexandria, EGY; 3 Modelling, Syreon Middle East, Alexandria, EGY; 4 Faculty of Pharmacy, Alexandria University, Alexandria, EGY; 5 Health Economics, Syreon Middle East, Cairo, EGY; 6 Health Economics, Syreon Research Institute, Budapest, HUN; 7 Center for Health Technology Assessment, Semmelweis University, Budapest, HUN

**Keywords:** health technology assessment, hta, algeria, roadmap, health technology assessment implementation

## Abstract

Background

The scarcity of resources restricts healthcare financing decisions, affecting the population’s health. Health technology assessment (HTA) balances restricted budgets with the best possible health outcomes. We aim to characterize the current status of HTA in Algeria and describe the future directions for HTA implementation according to the priorities set by local stakeholders.

Methods

Stakeholders from the public and private sectors responded to a policy survey about the current and preferred future status of HTA implementation in Algeria. The survey was administered during an online workshop and used a widely accepted international scorecard covering eight domains: capacity building, HTA financing, process and organizational structure, scope of HTA implementation, decision criteria, standardization of methodology, use of local data, and international collaboration. After that, one-on-one interviews with another local expert were conducted to validate and modify the draft recommendations. The interviewees were representatives from government agencies, hospitals, and pharmaceutical companies.

Results

Thirty-one experts filled out the HTA scorecard survey; most of them were from the public sector (74%). They highlighted that project-based HTA workshops or short courses were the most common form of HTA education in Algeria and recommended the establishment of postgraduate HTA training programs in the future to build sustainable capacities. They reported a lack of funding for HTA research and critical appraisal and recommended an increased public budget for HTA and the introduction of submission fees by manufacturers. There was consensus about the need for local HTA evidence generation in the future. Most of the experts advocated an explicit soft decision threshold. The interviewees further recommended using multi-criteria decision analysis in the short term. The application of quality indicators was believed to improve the reliability of the HTA process.

Conclusion

The results of our policy research delineate the gap between the current and preferred future status of HTA in Algeria based on insights from multiple stakeholders. The need to improve the educational HTA programs in Algeria, use local data in policy decisions, and increase funding for HTA were the most advocated recommendations.

## Introduction

Healthcare innovations are constantly evolving and introducing new and promising health technologies that benefit patients. These technologies generate a strong demand and diffuse across the healthcare system, which is the main driver of rising healthcare expenditure [1].

Innovative technology development has accelerated at an unprecedented rate [2]. The increasingly high price of effective medicines in areas with public health priorities (such as oncology) further threatens the sustainability of the healthcare system in all countries [3]. The growing number of medicines in areas with high unmet needs (such as rare diseases) and the emergence of genetic therapies will create a much greater challenge even for high-income countries to reimburse all new health technologies [4].

Although total health expenditures in Algeria have significantly increased during the last two decades [5], expanded delays have been reported about access to innovation [6]. Limited resources, combined with the increasing number of innovative health technologies, inevitably lead to difficult reimbursement decisions. Health technology assessment (HTA) can potentially improve the transparency and accountability of policy decisions in middle-income countries [7]. HTA is a multidisciplinary process that uses explicit methods to determine the benefits and value of health technologies. HTA aims to inform decision-making and promote an equitable, efficient, and high-quality health system by facilitating a more efficient allocation of resources [8,9]. Thus, it is expected that HTA may help enhance the efficiencies of healthcare systems.

In Algeria, pricing and reimbursement decisions are taken by centralized committees within the National Pharmaceuticals Agency and the Ministry of Labor, Employment, and Social Security (Laws). To date, there is no official HTA body at the national level. However, a sub-directorate of economic evaluation was established within the ministry in charge of the pharmaceutical sector.

For Algeria to implement HTA, a carefully designed process is required. Since HTA roadmaps may not be fully transferable to other jurisdictions, each country should develop a roadmap aligned with its objectives. The roadmap should consider the availability of human and financial resources and the country’s political, legal, and cultural aspects. Our study aims to contribute to a tailor-made HTA implementation roadmap in Algeria by assessing the gap between the current and the preferred future HTA environment.

## Materials and methods

Survey

We conducted a policy survey to describe the current environment of HTA implementation in Algeria and propose long-term objectives for HTA implementation. The survey utilizes an HTA implementation scorecard designed to support the formulation of HTA roadmaps in several countries [10]. The current and preferred future statuses of HTA implementation were explored in eight areas: capacity building, HTA funding, process and organizational structure, the scope of HTA, decision criteria, quality, and transparency of HTA implementation, use of local data, and international collaboration. As such, the scorecard served as the foundation for advising on the appropriate HTA structure and implementation process from the local stakeholders’ perspective.

The first, “HTA capacity building,” addresses the availability of well-trained experts for HTA supporting its implementation. The second domain, “HTA funding,” assesses the provision of financial support for HTA research and critical appraisal through public, private, or a mix of both. The third domain, “HTA legislation,” describes the role of HTA in the legal framework. The fourth domain, “Scope of HTA implementation,” identifies the different types of health technologies evaluated through HTA. The fifth domain, “Decision criteria,” specifies criteria for inclusion in the HTA process, like cost-effectiveness or budget impact analysis. Finally, the remaining domains are “The use of local data,” “Quality and transparency of HTA implementation,” and “International collaboration,” which address the need for local data in HTA, the quality and transparency measures in the HTA process, and the global exchange of reports and educational endeavors, respectively.

The survey included single-choice and multiple-choice questions, depending on the nature of the domain investigated. Participants consented that their survey responses could be aggregated and used anonymously in scientific publications. This survey was previously implemented in several countries, including Jordan, Egypt [11], Ukraine, Romania, and Turkey [12-15]. It was also implemented across regions like Latin America, the Middle East, and North Africa [10,16]. Therefore, using the same tool allows for the comparability of results. To improve accessibility and understanding, a field expert translated the survey from English to French. To ensure accuracy, backward translation was conducted. In the event of a discrepancy in the meaning after reverse translation, the translated survey was adjusted to ensure the meaning was preserved.

The selection of participants for this study was conducted through convenience sampling, ensuring the inclusion of individuals from a diverse range of institutions in Algeria who possess knowledge of HTA and are involved in the decision-making process regarding access to pharmaceuticals, including registration, pricing, reimbursement, procurement, and access policy. A target sample size of 30 survey respondents (with a bare minimum of 20 respondents) was proposed based on the adaptation of the same survey methodology in other countries.

An online workshop was held on March 22, 2022, with decision-makers representing different entities in the Algerian healthcare system. During the workshop, the concept of evidence-informed decision-making and details about HTA were presented. Toward the end of the workshop, the content of the survey was explained, and then the survey was electronically distributed through a proprietary platform.

Descriptive statistics using mean and standard deviation for continuous variables and frequency and percentage were used. Microsoft Excel 365 (Microsoft Corporation, Redmond, United States) was used for data analysis.

Survey results were then anonymously aggregated, and preliminary findings with main conclusions were reported as a list of draft recommendations by the research team. The developed recommendations were based on major identified gaps between the current and preferred statuses of HTA implementation concerning the eight domains.

Validation interviews

One-to-one interviews were conducted to validate and modify the draft recommendations. The interviewees were representatives from the Ministry of Health, the Ministry of Pharmaceutical Industry, the National Pharmaceuticals Agency, the Economic Committee, the Central Hospitals Pharmacy, the National Security Fund, hospital pharmacists, and pharmaceutical companies. The stakeholders involved in the one-to-one interviews were selected according to the same criteria as the survey participants, as detailed in the previous section.

At the beginning of the discussion, the project’s research objective and nature were introduced to stakeholders. We then presented the general structure of the survey and described the eight domains of HTA implementation included in the survey. Next, recommendations for each domain were assessed by the interviewees for the feasibility of their implementation. They were allowed to propose any additional idea or suggestion and whether they would recommend breaking down the implementation process into phases (short term within three years and long term from three to 10 years).

## Results

Survey results and validation

Demographics of Survey Respondents

From the total sample of 32 surveys, 31 were considered valid, as one respondent was non-Algerian. Twenty-two (74%) participants were employed in the public sector and five (26%) in the private sector. Around 84% of the respondents had primary education in pharmaceutical sciences. Further details, including the participants’ age and work field, are presented in Table 1. Responses to the survey in each domain are presented as percentages in Table 2, and the list of draft recommendations based on the survey responses is summarized in Table 3.

**Table 1:** Demographics of survey respondents‎ (n = 31) HTA, health technology assessment

Background information	Frequency (%)
Country	
Main employment	
Public sector	23 (74.2%)
Private sector	8 (25.8%)
Main employment – Public sector	
Field of work	
Decision-maker, policymaker, public payer, and Ministry of Health (potential HTA user)	11 (42.3%)
HTA agency	1 (3.8%)
Academic sector	8 (30.8%)
Public health care provider (e.g., clinician)	5 (19.2%)
Other	1 (3.8%)
Main employment – Private sector	
Field of work	
Health care industry (e.g., pharmaceutical or medical device company)	6 (75%)
Private health care provider (e.g., clinician)	0
Private health insurance	0
Consultancy	0
Journalist	0
Pharmaceutical trade sector (e.g., wholesaler and pharmacy)	1 (12.5%)
Other	1 (12.5%)
Demographic	
Major training	
Economics	1 (3.2%)
Pharmacy	26 (83.9%)
Medicine	1 (3.2%)
Other health care (e.g., nursing and dietetics)	0 (0.0%)
Multidisciplinary (at least two master’s degrees from the above list)	1 (3.2%)
Other	2 (6.5%)
Age	
Below 30	8 (25.8%)
Between 30 and 50	19 (61.3%)
Above 50	4 (12.9%)

**Table 2:** Aggregated results of valid responses from the HTA implementation survey (scorecard) HTA, health technology assessment; MCDA, multi-criteria decision analysis

Question	Current (current HTA status) n (%)	Preferred (aspired situation) n (%)
1. HTA capacity building		
a. Education		
No training	5 (16.1%)	0 (0.0%)
Project-based training and short courses	24 (77.4%)	3 (9.7%)
Permanent graduate program with short courses	0 (0.0%)	9 (29.0%)
Permanent graduate and postgraduate program with short courses	2 (6.5%)	19 (61.3%)
2. HTA funding		
a. Financing critical appraisal of technology assessment		
No funding for critical appraisal of technology assessment reports or submissions	27 (87.1%)	2 (6.5%)
Dominantly private funding (e.g., submission fees) by manufacturers for the critical appraisal of technology assessment reports or submissions	3 (9.7%)	14 (45.2%)
Dominantly public funding for critical appraisal of technology assessment reports or submissions	1 (3.2%)	15 (48.4%)
b. Financing HTA (i.e., HTA research)		
No public funding for technology assessment; private funding is not needed or expected	21 (70.0%)	2 (6.5%)
No or marginal public funding for research in HTA; private funding is expected	8 (26.7%)	6 (19.4%)
Sufficient public funding for research in HTA; private funding is also expected	0 (0.0%)	18 (58.1%)
HTA research is dominantly funded by public resources	1 (3.3%)	5 (16.1%)
3. Legislation on HTA		
a. Legislation on the role of the HTA process and recommendations in the decision-making process		
No formal role of HTA in decision-making	25 (80.6%)	1 (3.2%)
Dominantly international HTA evidence is taken into account in decision-making	3 (9.7%)	2 (6.5%)
International and additionally local HTA evidence is taken into account in decision-making	2 (6.5%)	20 (64.5%)
Local HTA evidence is mandatory in decision-making	1 (3.2%)	8 (25.8%)
b. Legislation on organizational structure for HTA appraisal		
There is no public committee or institute for the appraisal process	20 (64.5%)	1 (3.2%)
A committee is appointed for the appraisal process	8 (25.8%)	1 (3.2%)
The committee is appointed for the appraisal process with the support of academic centers and independent expert groups	0 (0.0%)	6 (19.4%)
A public HTA institute or agency is established to conduct a formal appraisal of HTA reports or submissions	1 (3.2%)	4 (12.9%)
Public HTA institute or agency is established to conduct a formal appraisal of HTA reports or submissions with the support of academic centers and independent expert groups	1 (3.2%)	13 (41.9%)
Several public HTA bodies are established without central coordination of their activities	1 (3.2%)	0 (0.0%)
Several public HTA bodies are established with central coordination of their activities	0 (0.0%)	6 (19.4%)
4. Scope of HTA implementation		
a. Scope of technologies (multiple choice)		
HTA is not applied to any health technologies	23 (74.2%)	1 (3.2%)
Pharmaceutical products	8 (25.8%)	29 (93.5%)
Medical devices	3 (9.7%)	24 (77.4%)
Prevention programs and technologies	0 (0.0%)	19 (61.3%)
Surgical interventions	0 (0.0%)	17 (54.8%)
Other scope of technologies (separated by commas)	0 (0.0%)	0 (0.0%)
b. Depth of HTA use in pricing and/or reimbursement decisions of health technologies		
HTA is not applied to any health technologies	20 (64.5%)	1 (3.2%)
Only new technologies with significant budget impact	11 (35.5%)	6 (19.4%)
Only new technologies	0 (0.0%)	4 (12.9%)
New technologies + revision of previous pricing and reimbursement decisions	0 (0.0%)	20 (64.5%)
5. Decision criteria		
a. Decision categories (multiple choice)		
None of the below categories are applied	14 (45.2%)	1 (3.2%)
Unmet medical need	6 (19.4%)	20 (64.5%)
Healthcare priority	7 (22.6%)	17 (54.8%)
Assessment of therapeutic value	7 (22.6%)	21 (67.7%)
Cost-effectiveness	4 (12.9%)	26 (83.9%)
Budget impact	10 (32.3%)	28 (90.3%)
Other decision categories	0 (0.0%)	1 (3.2%)
b. Decision thresholds		
Thresholds are not applied	29 (93.5%)	2 (6.5%)
Implicit thresholds are preferred	1 (3.2%)	10 (32.3%)
Explicit soft thresholds are applied in decisions	1 (3.2%)	16 (51.6%)
Explicit hard thresholds are applied in decisions	0 (0.0%)	3 (9.7%)
c. MCDA		
No explicit multi-criteria decision framework is applied	26 (83.9%)	3 (9.7%)
Explicit multi-criteria decision framework is applied	5 (16.1%)	28 (90.3%)
6. Quality and transparency of HTA implementation		
a. Quality elements of HTA implementation (multiple choice)		
None of the below quality elements are applied	23 (74.2%)	2 (6.5%)
Published methodological guidelines for HTA/economic evaluation	3 (9.7%)	21 (67.7%)
Regular follow-up research on HTA recommendations	2 (6.5%)	11 (35.5%)
A checklist to conduct a formal appraisal of HTA reports or submissions exists but is not available to the public	4 (12.9%)	8 (25.8%)
A published checklist is applied to conduct a formal appraisal of HTA reports or submissions	1 (3.2%)	11 (35.5%)
b. Transparency of HTA in policy decisions		
Technology assessment reports, critical appraisal, and HTA recommendations are not published	29 (93.5%)	3 (9.7%)
HTA recommendation is published without details of technology assessment reports and critical appraisal	1 (3.2%)	11 (35.5%)
Transparent technology assessment reports, critical appraisals, and HTA recommendations	1 (3.2%)	17 (54.8%)
c. Timeliness		
HTA submission and issuing recommendations have no transparent timelines	25 (83.3%)	2 (6.5%)
HTA submissions are accepted/conducted following a transparent calendar, but issuing recommendations has no transparent timelines	4 (13.3%)	18 (58.1%)
HTA submissions are accepted continuously and issuing recommendations has transparent timelines	1 (3.3%)	11 (35.5%)
7. Use of local data		
a. Requirement of using local data in technology assessment		
No mandate to use local data	13 (48.1%)	3 (9.7%)
The mandate of using local data in certain categories without the need for assessing the transferability of international evidence	5 (18.5%)	6 (19.4%)
The mandate of using local data in certain categories with the need for assessing the transferability of international evidence	9 (33.3%)	22 (71.0%)
b. Access and availability of local data		
Limited availability or accessibility to local real-world data	22 (71.0%)	1 (3.2%)
Up-to-date patient registries are available in certain disease areas, but payers’ databases are not accessible for HTA doers	7 (22.6%)	6 (19.4%)
Payers’ databases are accessible for HTA doers, but patient registries are not available or accessible in the majority of disease areas	1 (3.2%)	3 (9.7%)
Up-to-date patient registries are available in certain disease areas and payers’ databases are accessible for HTA doers	1 (3.2%)	21 (67.7%)
8. International collaboration		
a. International collaboration, joint work on HTA (joint assessment reports), and national/regional adaptation (reuse) (multiple choice)		
No involvement in joint work and no reuse of joint work or national/regional HTA documents from other countries	24 (88.9%)	1 (3.4%)
Active involvement in joint work (e.g., EUnet HTA Rapid REA, and full Core HTA)	1 (3.7%)	15 (51.7%)
National/regional adaptation (reuse) of joint HTA documents	1 (3.7%)	10 (34.5%)
National/regional adaptation (reuse) of national/regional work performed by other HTA bodies in other countries	2 (7.4%)	16 (55.2%)
b. International HTA courses for continuous education on HTA		
Limited interest in (1) developing/implementing and (2) participating in international HTA courses	9 (30.0%)	1 (3.2%)
Interest only in regular participation in international HTA courses	8 (26.7%)	5 (16.1%)
High interest in (1) developing/implementing and (2) participating in international HTA courses	13 (43.3%)	25 (80.6%)

**Table 3:** Draft recommendations based on major gaps between the current and preferred status of HTA implementation according to the eight domains of the scorecard survey HTA, health technology assessment; MCDA, multi-criteria decision analysis

Domain	Recommendations
Capacity building	More graduate and postgraduate HTA programs are recommended based on country-specific needs.
HTA funding	Public funding should be sufficiently increased for technology assessment and appraisal. The private budget for appraisal should be increased through submission fees to reach balanced funding for the HTA agency/agencies.
Legislation on HTA	Establishing a public HTA agency supported by academic efforts with major reliance on local HTA evidence. Alternatively, establishing multiple HTA agencies can also be considered with central coordination.
Scope of HTA implementation	Extending the scope of HTA from pharmaceuticals to non-pharmaceuticals is recommended in addition to revising previous policy decisions on top of evaluating new healthcare technologies.
Decision criteria	For the cost-effectiveness criterion, explicit soft thresholds should be used. In addition to cost-effectiveness and budget impact, several other criteria have to be considered when applying MCDA.
Quality and transparency of HTA implementation	Published methodological guidelines and checklists for critical appraisal are recommended to improve HTA work quality. In addition, the appraisal process should follow a clear timeline with flexible timelines for recommendations.
Use of local data	Development of multiple patient registries and utilization of local claims data (with the availability of an accessible electronic payer's database) are recommended.
International collaboration	Organizing and participating in international HTA courses is highly recommended, as well as involvement in joint HTA work and local adaptation of HTA work performed by other HTA bodies.

HTA survey domains

Capacity Building

**Figure 1:**
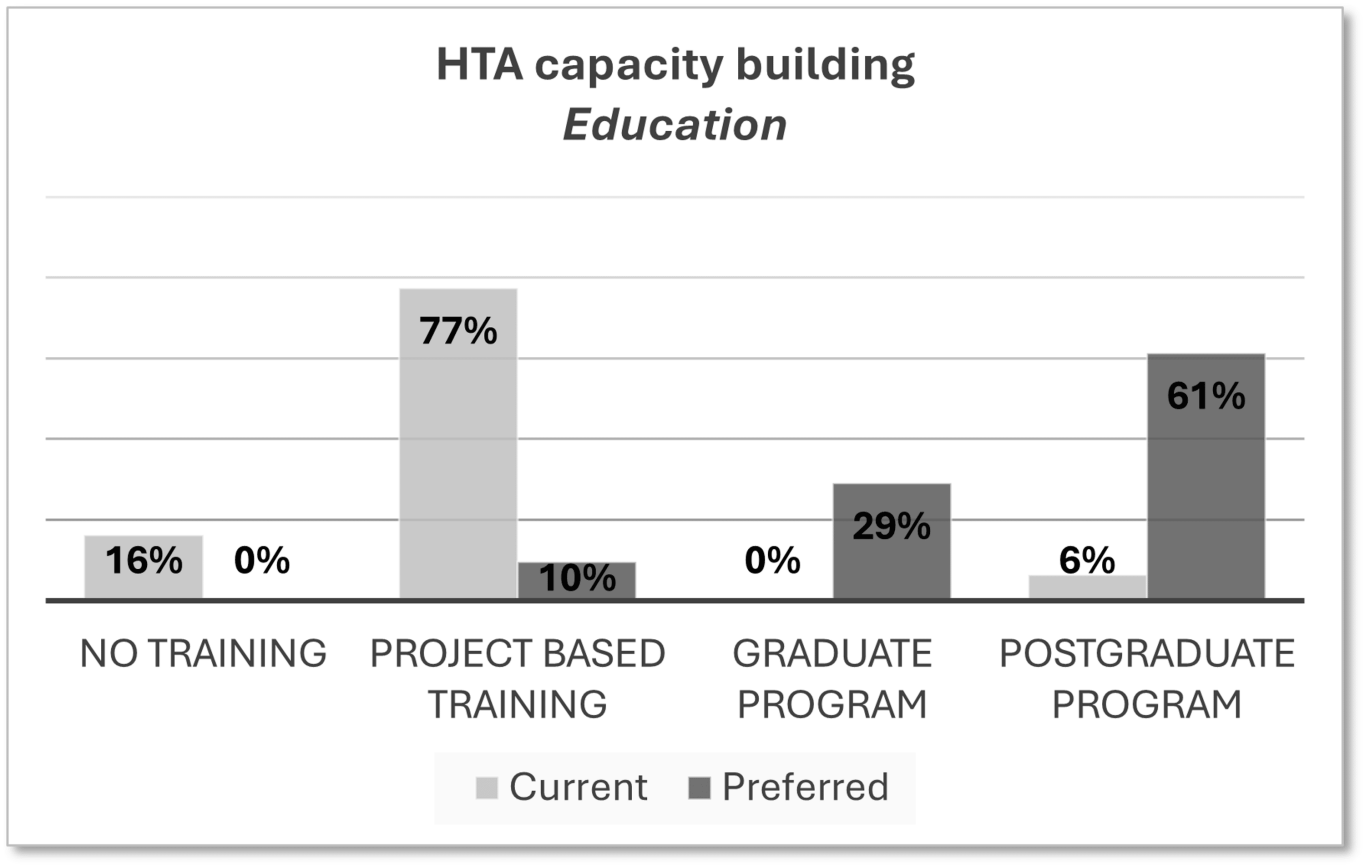
HTA capacity building current and preferred status HTA, health technology assessment

As HTA implementation requires highly skilled professionals, capacity building is critical for HTA roadmaps. Limited current options for HTA training were indicated by 94% of respondents, as shown in Figure 1. Project-based HTA workshops or short courses, usually sponsored by pharmaceutical companies, were the most common form of HTA education in Algeria, which may not be sufficient to induce hands-on training experience. In the future, most survey respondents preferred having permanent graduate and postgraduate programs in addition to the short courses already available.

During the one-to-one interviews, the interviewees confirmed that project-based HTA workshops or short courses were the only forms of HTA education in Algeria until the launch of the new master’s degree in pharmacoeconomics and market access within the Faculty of Pharmacy, University of Algiers, that has started the past academic year. They recommended expanding the postgraduate HTA training programs in the future to build sustainable capacities.

To implement the postgraduate HTA programs, the interviewees recommended training the trainers in the first phase through international collaboration and then developing HTA research and collaborating with health authorities to identify the gaps that need to be covered.

They also recommend maintaining short course programs for HTA users (such as pharmacists and physicians) and introductory undergraduate lectures for medical and pharmacy students. This will help to improve their general understanding of HTA [16].

HTA Funding

Sustainable funding is a crucial element of HTA implementation. Two phases of HTA implementation require funding. The first is the assessment phase, focusing on synthesizing scientific evidence and completing cost-effectiveness and budget impact analyses for health technologies. The second is the appraisal phase, concerned with validating the results of HTA dossiers and developing policy recommendations based on the main conclusions.

Most of the survey results (87%) indicated that currently, there was no funding for the critical appraisal of HTA evidence. However, in the future, the majority (94%) preferred significant funding for the critical appraisal phase, with respondents nearly evenly split between predominantly public (48%) and private (45%) funding sources, e.g., through submission fees paid by pharmaceutical companies.

For the assessment phase, limited current funding was also reported. Nevertheless, in the future, most experts (74%) preferred dominant or at least sufficient public funding. The interviewees confirmed insufficient public funding for technology assessment and appraisal. Although a sub-directorate for economic evaluation was established within the ministry responsible for the pharmaceutical industry, it faces a shortage of trained human resources.

Sufficient funding was recommended for both HTA research and critical appraisal. The interviewees indicated that a sufficient public budget should be allocated to start the appraisal activity, fees should then be applied in the second phase, and private funding should become increasingly important.

They recommended that data collection and evidence generation in the reimbursement submission dossier should be the responsibility of the manufacturers. Furthermore, they agreed that a dedicated HTA body should be put in place in the long term with sustainable funding from both public and private sources.

Legislation on HTA

Capacity building and sufficient funding are essential for implementing HTA. Nonetheless, it is imperative to recognize that the actual utility of HTA is significantly undermined if it is not involved in the decision-making process.

Regarding the role of HTA in the decision-making process, 81% of the respondents reported that, currently, HTA has no formal role in the decision-making process. At the same time, 10% of the respondents acknowledged that the international HTA evaluation reports were being considered. On the other hand, almost 90% of respondents indicated a need to change this practice. Instead of relying dominantly on international HTA evidence, increasing the role of local HTA evidence or even mandating its use in policy decisions is recommended in the future.

For the organizational structure, 65% of the respondents indicated that they were not aware of any public committee or institute responsible for the appraisal process, and 26% indicated that there was a committee appointed for the appraisal process, which reflected the non-transparency of how evidence has been taken into account in the current decision-making process.

In the future, some respondents (19%) would still prefer establishing a committee for the appraisal process with the support of academic centers and independent expert groups. On the other hand, most respondents (56%) preferred a national HTA agency with or without academic support. At the same time, 19% opted for several HTA agencies with or without central coordination to boost HTA implementation.

The interviewees indicated that there has been a formal role for HTA regarding the pricing process since December 2020, but it has not been clearly defined. They added that the respondents may not be aware of the new pricing process set by the ministerial order of December 20, 2020, where economic and pharmacoeconomic studies are one of the parameters that can be used to set the pharmaceutical prices by the economic committee [17]. They also mentioned the economic evaluation sub-directory in charge of pharmacoeconomic studies assessment [18].

The interviewees reported that the Reimbursement Committee relied dominantly on international HTA evidence, especially medical services rendered by the French National Authority for Health (Haute Autorité de Santé). They all agreed that HTA required further development, with a clear delineation of its role in the pricing and reimbursement processes.

Scope of HTA Implementation

The scope of HTA implementation focuses on the range of assessed health technologies and related factors.

Based on the survey results, 74% of the respondents reported that HTA was not applied to any health technologies, while 26% reported that HTA was utilized to support decisions related to pharmaceuticals. In the future, most respondents preferred expanding the scope of HTA to different technologies, including pharmaceuticals (94%), medical devices (77%), prevention programs (61%), and surgical interventions (55%).

**Figure 2:**
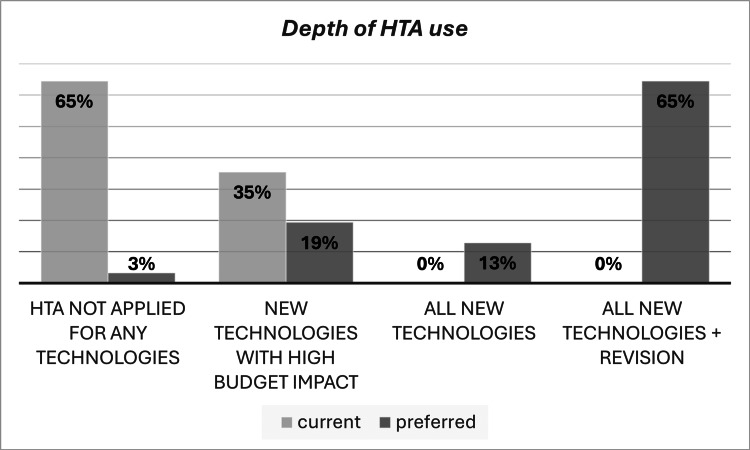
Depth of HTA use current and preferred status HTA, health technology assessment

Almost 36% of the respondents indicated that HTA was being used for only new technologies with significant budget impact, but 65% believe that, in the future, the HTA role should be extended to cover all new technologies and for the revision of previous pricing and reimbursement decisions, as shown in Figure 2.

Based on the interviewees’ discussions, it was recommended to expand the use of HTA from the new pharmaceuticals with a high budget impact on registration to the revision and medical devices in the short term (three to five years) and then to expand the use of HTA to other technologies, such as prevention programs, within six to 10 years.

Decision Criteria

HTA can embrace multiple criteria in decision-making; however, countries may not necessarily consider all criteria in their policy process. According to 45% of the respondents, no criteria were explicitly considered in Algeria. Thirty-two percent of survey respondents mentioned budget impact, while 23% advocated healthcare as a priority. In the future, respondents preferred considering more categories for decision-making, including therapeutic value (68%), cost-effectiveness (84%), budget impact (90%), and unmet medical need (65%).

**Figure 3:**
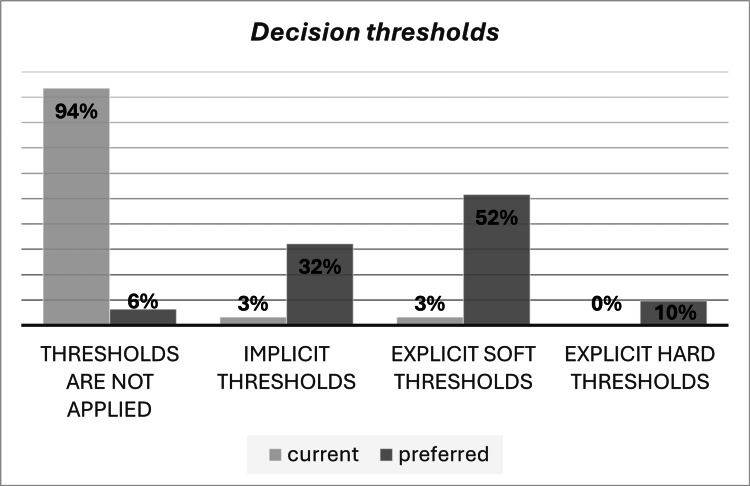
Decision thresholds current and preferred status

As shown in Figure 3, 29 (94%) survey respondents indicated that cost-effectiveness threshold(s) was not employed in Algeria. The same number of respondents (94%) preferred the adoption of a decision threshold in the long term, of which 61% preferred using an explicit one. More than 52% of the respondents preferred soft thresholds to allow the possibility of reimbursing exceptional priority medicines without cost-effectiveness evidence. Nevertheless, 32% still preferred an implicit threshold.

The multi-criteria decision analysis (MCDA) framework is currently applied only in a few cases. Respondents, however, 90% advocated its use and implementation.

Based on the interviews, the decision-making process for pricing today considers multiple criteria without properly using MCDA as a tool. Budget impact and unmet medical needs were the most commonly reported criteria. Interviewees recommended using MCDA in the short term by weighing the most important criteria for public health in Algeria.

Using cost-effectiveness analysis besides the budget impact analysis, which has just started to be used, was also recommended. A number of interviewees recommended the use of quality-adjusted life years in the long term. Unfortunately, no cost-effectiveness threshold is currently applied in Algeria, but the interviewees recommended adopting an explicit threshold in the long term. Also, they advocated a soft, explicit threshold to allow access to specific lifesaving technologies in specific areas, such as rare diseases.

Quality and Transparency of HTA Implementation

Although multiple approaches can improve the quality of HTA, 74% of survey respondents were unaware of applying such tools for quality improvement. In the future, however, most respondents (68%) preferred publishing methodological guidelines for HTA/economic evaluation to standardize the appraisal process. Regular follow-up research on HTA recommendations and published checklists are applied to the formal appraisal of HTA reports or submissions, both preferred by 11 (35%) survey respondents.

Ninety-four percent of the respondents reported that HTA reports and recommendations were inaccessible to the public. More than 90% preferred changing this practice, 35% recommended publishing HTA recommendations only, and 55% believed that technology assessment reports, critical appraisals, and HTA recommendations should be published in the public domain.

Regarding HTA timelines, 83% of respondents reported limited transparency for HTA timelines. Therefore, 94% of respondents advocated that HTA submissions should have transparent timelines in the future, of which 35% preferred that HTA submissions be accepted continuously and that issuing the recommendations aligns with transparent timelines. Still, 58% preferred to keep issuing recommendations loose without transparent timelines.

The interviews showed an agreement on the importance of transparency. In the short term (three to five years), the interviewees recommended issuing methodological guidelines. They preferred publishing the recommendations and appraisal reports once the methodological guidelines were issued. They mentioned that timelines should clearly be set.

Use of Local Data

Half of the survey respondents (48%) indicated that local data were not mandated in the current HTA process. In the future, 71% of survey participants preferred mandating the use of local data for HTA evidence and evaluating the transferability of international HTA evidence when the required local information is not available. Limited availability or accessibility to local real-world data was reported by 71% of respondents. Devoting more resources to building patient registries or payer databases was recommended by 77% of respondents.

Interviewees highlighted the value of local healthcare data during their discussions. The representatives of the ministry in charge of the pharmaceutical industry pointed out that economic models, including local data, just started to be required from manufacturers for pricing. Furthermore, they indicated the scarcity of local data and the need to construct local databases and to allow access to payers’ available databases, such as the health insurance fund database “Chifa.” The interviewees specified that the mandatory use of HTA will create a new dynamic in collecting healthcare data in Algeria. Whether in the short or long term, experts agree that international evidence had a place in the process in the condition of use transferability. Additionally, in the long term (six to 10 years), the interviewees recommended developing robust databases. Most interviewees recommended establishing a central HTA body, except one who suggested establishing several HTA bodies within the hospitals.

International Collaboration

Most respondents (89%) reported limited involvement in joint international work. However, active involvement in joint work initiatives or the reuse of HTA materials prepared by international HTA bodies was preferred by almost all respondents. International collaboration can also contribute to the capacity-building process through participation in international HTA courses, as preferred by almost all respondents (97%).

Based on stakeholders’ discussion, collaboration with other countries in the region through involvement in joint work could be useful to avoid duplicating efforts, but local adaptation is necessary in this case.

Summary of proposed recommendations by stakeholders

Based on stakeholders’ discussions, an action plan with clear timelines was developed. Table 4 describes specific actions needed in each domain to implement HTA fully.

**Table 4:** Actions for HTA implementation HTA, health technology assessment; MCDA, multi-criteria decision analysis; QALY, quality-adjusted life year

Element	Action within three years	Actions from three to 10 years	Challenges and strategies to overcome them
Capacity building	Train the trainers. Expand postgraduate programs. Maintain short courses and undergraduate introductory courses	Developing research programs. Collaboration with health authorities regarding specific technologies	Challenge: Significant investment in training, resources, and infrastructure. Strategy: Collaborate with international HTA organizations for experts and seek funding from governmental and non-governmental organizations to support educational initiatives.
HTA funding	Evidence submission funded by industry. Public funding for appraisal	Submission fees. Dedicated HTA body. Public/private funding	Challenge: Budgetary constraints and competing priorities. Strategy: Advocate for HTA’s value in improving healthcare efficiency to policymakers and stakeholders. Implement obligatory submission fees for manufacturers to create a revenue stream for HTA activities.
Legislation on HTA	Clearly identify the role of HTA within the pricing and reimbursement processes.	Establish a dedicated HTA organization.	Challenge: Political will and legislative changes are required. Moreover, capacities are needed to undertake the required HTA activities. Strategy: Engage with policymakers to demonstrate the benefits of HTA. Form alliances with healthcare professionals, patient groups, and academic institutions to build a strong case for legislative support. Work on building capacities through partnerships and educational programs.
Scope of HTA implementation	New pharmaceuticals with high budget impact. Revision medical devices	Full scope (prevention programs)	Challenge: Additional expertise and resources are needed. Also, funding will be required to be increased. Strategy: Prioritize high-impact areas first and gradually expand the scope of HTA. Train existing staff in new areas and recruit specialists as needed. Expand the funding pool.
Decision criteria	Budget impact and cost-effectiveness. Explicit soft threshold MCDA	Cost-utility (QALY)	Challenge: Can be complex and resource-intensive. Strategy: Leverage existing international MCDA frameworks and adapt them to the local context. Provide training to decision-makers on the use of these frameworks to ensure consistency and transparency. Also, develop local MCDA frameworks for high-priority unmet needs such as off-label medicines.
Quality and transparency of HTA implementation	Methodological guidelines. Publish appraisal recommendations. Set timelines.	Publish appraisal reports	Challenge: Requires systematic changes, continuous monitoring, and more capacities. Strategy: Develop clear methodological guidelines in collaboration with international experts. Implement an online platform for publishing HTA reports and recommendations to enhance transparency.
Use of local data	Allow access to payers databases.	Construct robust databases.	Challenge: Can be hindered by the lack of robust data infrastructure and limited access to existing data sources. Strategy: Invest in the development of patient registries, data digital infrastructure, and electronic health records. Establish partnerships with healthcare providers and insurers to facilitate data sharing and access.
International collaboration	Reuse of material with adaptation	Involvement in joint work	Challenge: Limited existing involvement in international HTA collaborations and lack of international networking. Strategy: Actively seek partnerships with international HTA bodies and participate in global HTA initiatives. Encourage the adaptation of international best practices to fit local needs through pilot projects and phased implementation.

## Discussion

Despite the improvement in health indicators, the Algerian health system is characterized by mismanagement, especially within hospitals [19]. In parallel, health expenditures in Algeria are continually increasing due to demographic and epidemiological factors; the country still experiences limited access to medical innovations. Consequently, enhancing effectiveness within the health system is necessary [19,20].

Amid growing economic constraints, the efficient allocation of limited healthcare resources has taken on heightened significance. By comparing costs and outcomes associated with health interventions, HTA emerges as a potent instrument, aiding decision-makers in reaching logical and informed choices [21].

Despite the patterns of HTA roadmaps described in the scientific literature, no single HTA roadmap could fit all countries. HTA roadmaps have limited transferability as they consider factors like the country’s size, gross domestic product per capita, public health priorities, social values, and healthcare financing systems [22].

Similar to other countries in the Middle East and North Africa (MENA) region, health policy initiatives were taken to implement HTA in Algeria. Therefore, a roadmap is needed to guide decision-makers.

In the MENA region, there is a growing interest in HTA implementation [16]. This is due to the increasing cost of healthcare and the need to ensure that resources are used efficiently. As a result, some middle-income countries in the region have applied the same scorecard in similar policy surveys, such as Egypt [11] and Jordan [12]. Our results show some similarities with the findings from the scorecards of Egypt and Jordan in certain aspects and some variations in the specifics. Given that Algeria's scorecard is comparable to those of Egypt and Jordan, we will focus on the discrepancies.

The gap between the current and preferred future status of HTA implementation in Algeria can stem from the fact that HTA implementation is at its very early stage in Algeria and the MENA region.

In Algeria, there was consensus about raising capacity building through postgraduate programs besides short courses. As well as to maintain the introductory undergraduate courses in health economics for pharmacy and medicine students to enhance their understanding of the discipline. Despite the implementation of a new post-graduation degree in market access and pharmacoeconomics, a gap remains in HTA training and education between the current and preferred status It stems from the limited human capacities available and the lack of long-term investment in HTA education infrastructure. These results are in accordance with the findings of other researchers from the region [12,16].

The respondents highlighted the insufficient funding for HTA research and the critical appraisal. Public and private funding of the appraisal process is preferred. The primary reason for the funding gap is budgetary constraints and the limited financial prioritization of HTA activities. The public funding will indicate the political will for HTA implementation. However, sustaining the appraisal of HTA submissions relying on public funding can be questionable. So, private funding is also required. For HTA research, dominant, or at least sufficient, public funding is recommended.

According to the survey respondents, HTA had no formal role, but the interviewees confirmed that HTA now has a formal role within the pricing process. Nevertheless, this role remains unclear in informing health policy decisions in Algeria. The survey respondents were probably unaware of the new regulation that recently came into effect regarding the pricing procedure and the possibility of using HTA as one of the criteria to consider. Although HTA now has a formal role within the pharmaceutical pricing process, both respondents and experts emphasized the need for a clearer definition of this role in the future and the expansion of HTA’s involvement in the reimbursement process. The primary reason for this gap is the resistance to change within existing processes, as external reference pricing is traditionally used by the economic committee for pricing and the reimbursement committee relies on HTA evidence from other countries for reimbursement decisions. Some existing HTA activities rely on evidence from other countries, especially regarding reimbursement. They suggested that more local evidence should be generated and used in future HTA endeavors. Moreover, the respondents emphasized the importance of having a national HTA agency coordinate and oversee the HTA process. Although such an agency does not exist yet in Algeria, there are some signs of progress, such as establishing a sub-directorate for economic evaluation in charge of conducting appraisals and sharing recommendations with the existing centralized committee for pricing decisions. These may indicate a political interest in HTA and facilitate the development of local HTA capacity.

The organizational structure of HTA can influence the efficiency and effectiveness of the process. Fortunately, in Algeria, where the healthcare system is centralized, the respondents recommended a single public HTA agency to conduct HTA appraisals. This approach differs from that of Jordan’s experts, who preferred multiple public HTA bodies, reflecting the fragmented nature of their healthcare system. In Egypt, the survey respondents supported having multiple public agencies, but some interviewees argued for a single HTA agency to prevent overlapping tasks and bias. However, by the end of the discussions, the experts in Egypt reached a consensus that multiple agencies with central coordination would be more politically feasible [12,16]. The difference in healthcare system structure can be the cause of this discrepancy. In Algeria, the healthcare system is centralized, and there is compulsory, unique public health insurance.

Experts in Algeria recommended a gradual implementation of the HTA rather than an immediate, full-scale implementation. This approach allows capacity building, legislation, and financing to develop sustainably. The limited application of HTA beyond pharmaceuticals with significant budget impacts is attributed to resource constraints and a lack of expertise. A similar approach has been adopted in countries such as Jordan, Tunisia, and Saudi Arabia, where the initial focus is on high-budget innovative pharmaceuticals, with the scope and depth of HTA implementation expanding thereafter [16].

According to some of our respondents, decision-making in Algeria currently takes into account budget impact analysis and healthcare priorities but lacks clear decision criteria. For future HTA activities, the respondents agreed on the importance of having decision criteria, especially budget impact, cost-effectiveness, therapeutic value, and unmet medical needs. Most of the respondents suggested adopting explicit thresholds, preferably soft thresholds. Soft thresholds are more flexible than hard thresholds and can accommodate the reimbursement of some therapeutic categories that would otherwise be excluded, such as orphan drugs [23].

The vast majority of respondents indicated the limited application of the MCDA framework, as confirmed by the interviewees. They all recommended increasing its use. MCDA can help decision-makers purchase high-cost off-patent pharmaceuticals [24] and medical devices [25].

The respondents indicated that the current HTA process in Algeria does not apply any quality elements. Thus, they suggested developing methodological guidelines for HTA to enhance the quality of the HTA process. They also pointed out that HTA reports are not published, including assessments, recommendations, and critical appraisals. Thus, they proposed that these reports should be made public in the future to enhance transparency. Regarding the timelines, the respondents stated there were no transparent timelines for issuing the recommendations for HTA submissions. They recommended setting transparent timelines for the HTA submissions, but not for the recommendations. This recommendation contrasts with the findings from Egypt and Jordan, where the respondents supported having transparent timelines for both the HTA recommendations and the submissions. The difference in capacities and workload can be the cause of the discrepancy. Timeliness for issuing recommendations is essential for achieving the benefits of HTA. Delayed assessment may result in unequal access [26] to healthcare and hurdles to the pharmaceutical industry [27], which may affect the healthcare system negatively. The respondents may have aimed to ease the burden on the public HTA agencies, especially in the initial stages of implementation and with limited capacity, but the absence of transparent timelines for issuing recommendations may cause more problems.

Until recently, using local data for HTA was not mandated in Algeria; however, the sub-directorate of economic evaluation has just started asking for economic models, including local data. The limited use of local data is a result of the scarcity of local data infrastructure and restricted access to existing payer databases. The interviewees seemed aware of the scarcity of local data, but they believe making it mandatory will help generate readily available and high-quality local data. Given the scarcity of local real-world data, the respondents recommended establishing patients’ registries and facilitating access to payers’ databases. In this context, it is noteworthy that utilizing high-quality evidence on relative treatment effectiveness from other countries may conserve resources for HTAs [28]. Although specific components of HTA reports may be transferable [29], and so international evidence can provide a helpful starting point in local value judgment, it is crucial to adapt them to local data and context. Relying solely on international HTA recommendations to drive local decisions, especially regarding cost considerations, often with confidential components, could lead to inappropriate and harmful policies [30]. Hence, transferring global HTA knowledge should be approached carefully [31].

The respondents revealed the absence of involvement in joint work with HTA agencies in other countries. They recommended actively engaging in joint work with international agencies and reusing the work performed by other HTA bodies in other countries. Also, the respondents recommended a capacity-building process through participation in international HTA courses. International collaboration helps avoid duplication of efforts. Also, joint assessment may help reduce the service burden (especially in cases of low capacity) and reduce the time needed for assessment.

Our HTA roadmap provides an action plan with clear timelines for implementing HTA. Furthermore, designing a roadmap is just one step in HTA implementation. Continuous monitoring of actions is recommended to allow for readjustment of timelines or even changes to certain action items.

The process of HTA and its implementation will contribute to establishing a balance between equity, quality healthcare, and efficiency of decisions. It will also support resource allocation decisions regarding the reimbursement of health technologies. Furthermore, establishing a formal and institutionalized system of HTA is needed to implement the proposed recommendations and monitor for any readjustments [32].

Limitations

Our study has some limitations, including the relatively small sample size; however, this was managed by the involvement of experts on the topic. Our sample did not include patients' representatives owing to the lack of understanding of the HTA principles by the patient associations in Algeria. Furthermore, a convenience sampling technique was utilized to select participants for the survey, which may limit the generalizability and representativeness of our findings. Also, we used a self-administered online survey to collect data, which may introduce response bias and social desirability bias.

Despite these limitations, our study provides valuable insights into the current status and future perspectives of HTA implementation in Algeria. The recommendations from this study provide chronologically specific actions for full HTA implementation and should act as a supportive tool for decision-makers to fully implement HTA in Algeria in the long term.

## Conclusions

Implementing HTA in Algeria requires collaboration from different sectors and stakeholders. The more aligned the action plan is with Algeria’s capabilities, the more comprehensive the impact on the health system. The implementation of the action plan is expected to make the health system more efficient, placing priorities on accessing effective health technology for patients. The roadmap functions as a strategic instrument for evaluating the advancement of HTA implementation.
